# Baseline Characteristics, Case Mix, and Outcomes of Patients Admitted to a Busy Urban Acute Medical Unit in Ireland

**DOI:** 10.7759/cureus.94167

**Published:** 2025-10-08

**Authors:** Khalid Elamin, Adam H Dyer, Keneilwe Malomo, Declan Byrne, Joseph Browne, Ontefetse Ntlholang

**Affiliations:** 1 Cardiology, Royal Free Hospital, London, GBR; 2 Geriatrics, St James’s Hospital, Dublin, IRL; 3 Geriatrics, General Medicine and Stroke, St James's Hospital, Dublin, IRL

**Keywords:** acute hospitals, acute medical admissions unit, case-mix, presenting complaints, service provision

## Abstract

Background

Acute medical admissions units (AMAUs) have been associated with decreased mortality and length of stay (LOS) for acute unscheduled hospital admissions. However, the needs and demands of patients admitted to AMAUs are constantly evolving in response to demographic changes and variations in the prevalence of illnesses.

Method

All patients admitted to the AMAU of a single-center hospital in Ireland in 2017, pre-COVID-19 era, were included in this study. Routine demographic information, including age, gender, date of admission, and duration of admission, and data regarding presenting complaints and immediate outcomes were collected using the hospital’s patient administration system and electronic patient records.

Results

Of 3,037 admissions, 1,515 (49.9%) were female, the mean age was 61.7±20.2 years, and 971 (32.0%) were aged 75 years or older. The most common categories of admission were respiratory (719 patients, 23.7%), neurology (539 patients, 17.7%), falls and impaired functional status (314 patients, 10.3%), cardiovascular (278 patients, 9.5%), and clinical toxicology (209 patients, 6.9%). The most common reasons for admission were falls, syncope, and collapse (283 patients, 9.32%), lower respiratory tract infection (242 patients, 8%), exacerbation of chronic obstructive pulmonary disease (206 patients, 6.8%), drug overdose (112 patients, 3.7%), and urinary tract infections (106 patients, 3.5%). The median LOS was five days (IQR: 2-12), and 317 patients (10.4%) had a prolonged LOS (defined as 30 days or greater).

Conclusion

This study provides a detailed case mix in terms of presenting complaints and outcomes across all age groups. The data are of paramount importance regarding the provision of service, the ongoing structuring of acute hospitals, the mix of skills needed across AMAUs, and the guidance that training bodies provide to educate workers in acute medicine.

## Introduction

A consensus statement by the Royal College of Physicians defines the specialty of acute medicine as the “immediate and early specialist management of acutely ill patients suffering from a wide range of medical conditions requiring urgent or emergency care” [1]. Acute medicine has grown rapidly as a specialty in recent years and has adapted to ever-evolving needs and demands associated with changes in demographics and the prevalence of various illnesses. Defining these clinical demands “at the front door” remains critical.

One particularly welcome development in acute care is the establishment of acute medical admissions units (AMAUs) to deliver consistently high-quality care. The AMAU in our hospital was established in 2003 and has reported significant benefits in terms of short- and long-term outcomes [2-6]. The establishment of AMAUs has been associated with decreased mortality and length of stay (LOS) for acute unscheduled hospital admissions and emphasis on a focused and high-volume approach to acute admissions [7,8].

Many standard data relating to unscheduled inpatient admissions are based on clinical coding that is usually assigned by administrative staff following the end of an acute medical admission. While such data are a valuable indicator of the prevalence of various conditions and in-hospital morbidity and mortality, and, indeed, may serve to model the costs associated with an unscheduled admission, they may not accurately reflect the initial presenting complaints or symptoms on arrival. The presenting complaint drives much of the initial investigation and treatment in patients admitted to an acute hospital. Thus, the exact case mix typically seen at the front door remains unclear.

Defining the initial case mix in terms of presenting complaints and suspected diagnoses is crucial for understanding the clinical workload of AMAUs and, therefore, the skill mix needed in these units and for designing educational curricula to implement changes in practice, such as establishing specific clinical pathways for common illnesses. To help fill this gap in the literature, in the present study, we reported the baseline characteristics, detailed information on the case mix based on initial presenting complaints, systems affected, and the immediate outcomes of all patients admitted to an AMAU in Ireland over one year.

## Materials and methods

Study setting

Our institution, located in Dublin, Ireland, is a large urban tertiary referral facility with a catchment population of approximately 270,000 people. It is located in an inner city, and this area has a high deprivation rate [9]. Adults presenting to the emergency department (ED) are initially assessed and triaged by ED staff. The AMAU in the hospital was established in 2003, and other studies have described its operation [2-6]. Briefly, five AMAU consultants are responsible for the acute medical team on the general rota. Their skill mix includes stroke, geriatrics, respiratory care, cardiology, rheumatology, infectious disease, and falls and blackout prevention. They meet in the morning to distribute the patients admitted equally among the five consultant teams. In addition, dedicated referral pathways have been established in the ED for specific acute medical illnesses, including stroke (covered by geriatrics/neurology) and ST-segment elevation myocardial infarction (STEMI) (cardiology). In addition, the geriatric staff in our hospital provides various services through Mercer's Institute for Successful Ageing, including consulting services, a day hospital for managing admissions, an on-site nursing home, and on-site rehabilitation facilities. Furthermore, they admit four patients daily on weekdays and three patients daily on weekends who would benefit most from their input, depending on bed capacity. There are also dedicated direct referral pathways for hematology and oncology patients and for surgery and psychiatry.

Data collection and participants

All patients admitted to the AMAU from January 1 to December 31, 2017, were included in this study's analysis. An Excel sheet (Redmond, WA: Microsoft Corp.) was used to record the data. Data related to the date of admission and discharge were obtained from the hospital’s patient administration system and the electronic patient records, which are linked. Routine demographic information was also obtained through these systems. For this study, the main reason for each patient’s admission was recorded by the consultant on take at the post-take AMAU morning meeting mentioned above. When multiple reasons for admission were recorded, the most severe complaint or the reason most pertinent to the initial investigation and management of the patient was used for the analysis. In practice, this typically prioritized the most acute, potentially life-threatening presentation (e.g., chest pain over minor trauma). This pragmatic, clinician-led approach ensured consistency in categorizing the primary presenting complaint across the cohort. These data were recorded electronically for every meeting in 2017. The main reasons for admission were then coded into categories based on the system affected (e.g., cardiovascular, respiratory, or gastroenterology) and specific problem (e.g., chest pain, shortness of breath, or arrhythmia). To ensure consistency with the previous literature, we constructed the categories and diagnoses based on a previously published study of the case mix of a busy acute medical unit [10].

Data analysis

The data were collated and fully anonymized. The mathematical analysis was performed using STATA IC v15 software (College Station, TX: StataCorp LLC). The descriptive parametric data were reported as means (and standard deviations), while the non-parametric data were used as medians and interquartile ranges. To assess the case mix of the patients attending the AMAU, the percentages in the discrete categories were reported as percentages of the overall total. For the outcome analysis, the LOS was calculated as the time from the first admission under the care of an AMAU consultant to the time of discharge from the hospital (consistent with routinely completed discharge summaries).

## Results

Baseline characteristics

**Figure 1:**
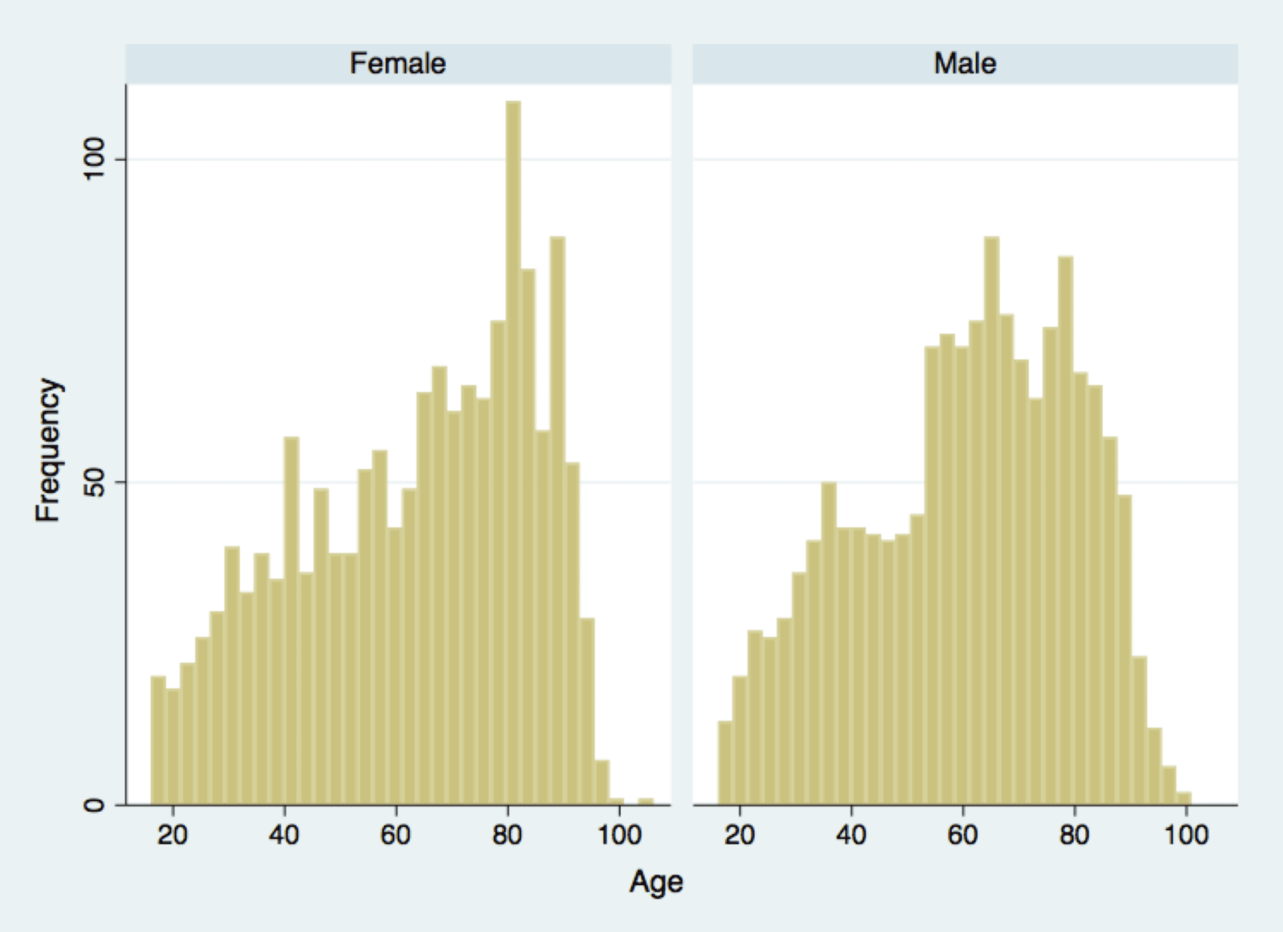
Age profile of patients admitted to the acute medical admissions unit by gender.

**Figure 2:**
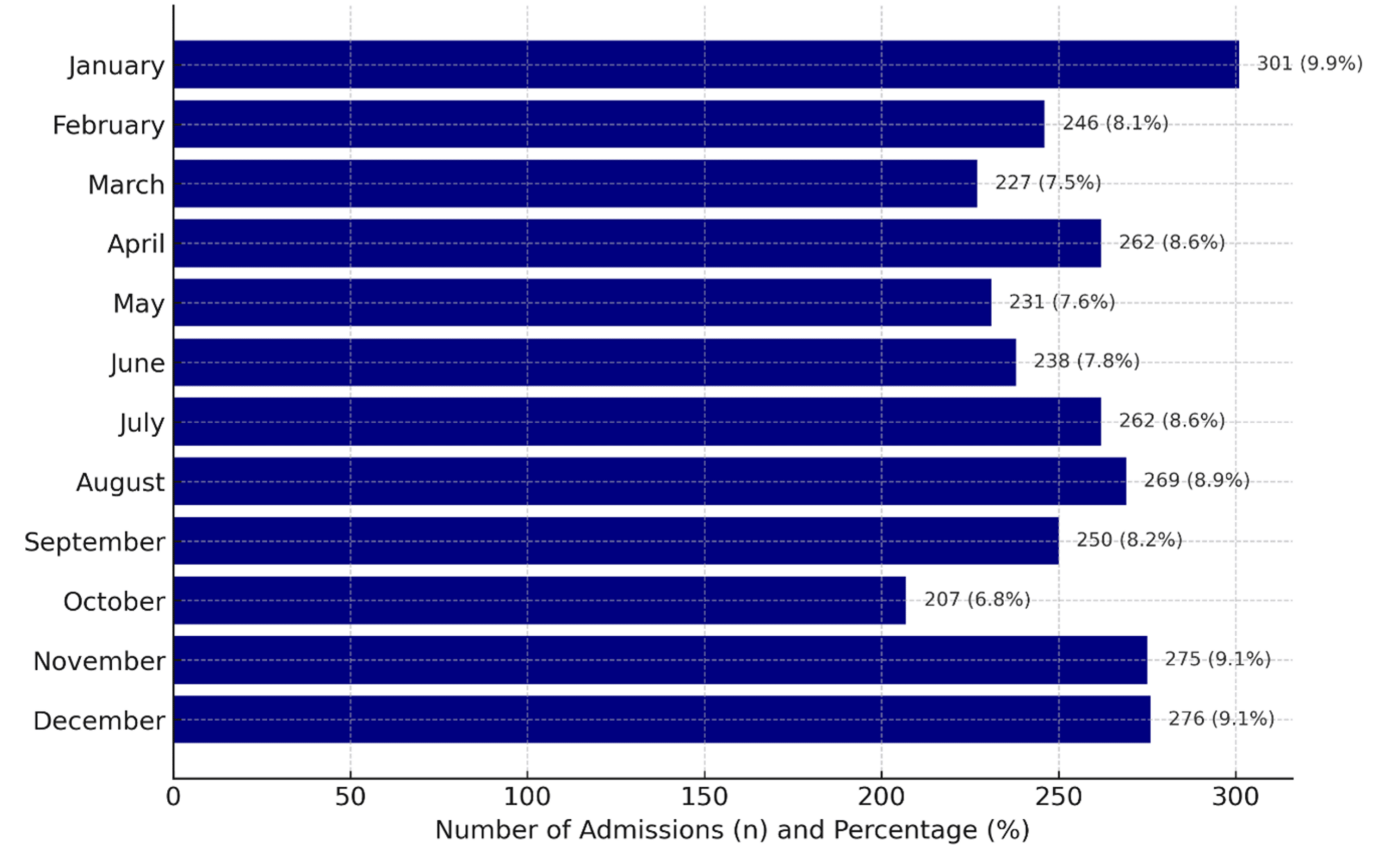
Admissions per month to the acute medical admissions unit.

Data were available for the entire cohort. A total of 3,037 patients were included, 1,515 (49.9%) of whom were female and were admitted to the AMAU during the study period. Their mean age was 61.7 years (SD=20.2; range=16-106 years). The age distribution by gender is shown in Figure 1. A total of 971 patients (31.97%) were aged 75 years or older. The mean number of admissions per month was 253 (SD=25.8), with the maximum number of admissions (n=301) in January and the minimum number in October (n=207) (Figure 2).

Reasons for admission

**Figure 3:**
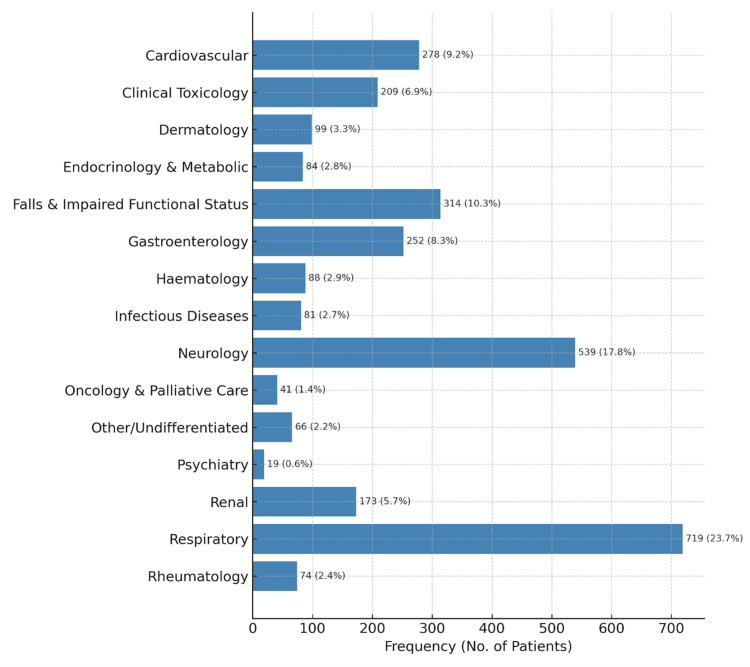
Major disease categories of admissions to the acute medical admissions unit.

**Figure 4:**
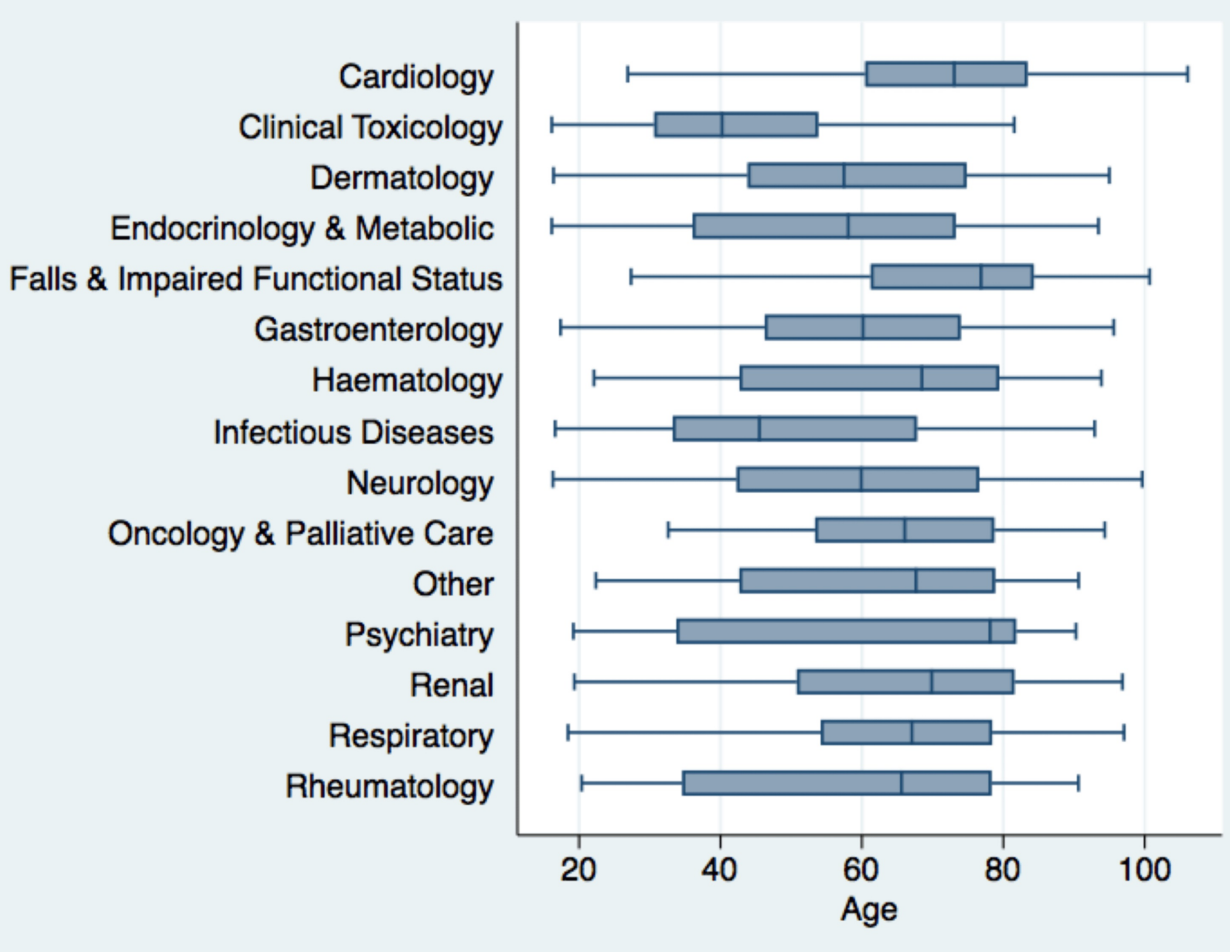
Age distribution by major disease categories.

The primary reason for admission (i.e., the presenting complaint) was prospectively recorded for each patient to define the case mix of those admitted to the AMAU. The most common category of admissions (major disease category) was respiratory, accounting for 23.68% of all admissions (n=719). Neurology (n=539, 17.75%), falls and impaired functional status (n=314, 10.30%), cardiovascular (n=278, 9.15%), gastrointestinal (n=252, 8.30%), and clinical toxicology (n=209, 6.88%) were the other common categories of admission, each accounting for more than 200 admissions per annum (Figure 3). The age distribution by major disease category is presented in Figure 4.

**Figure 5:**
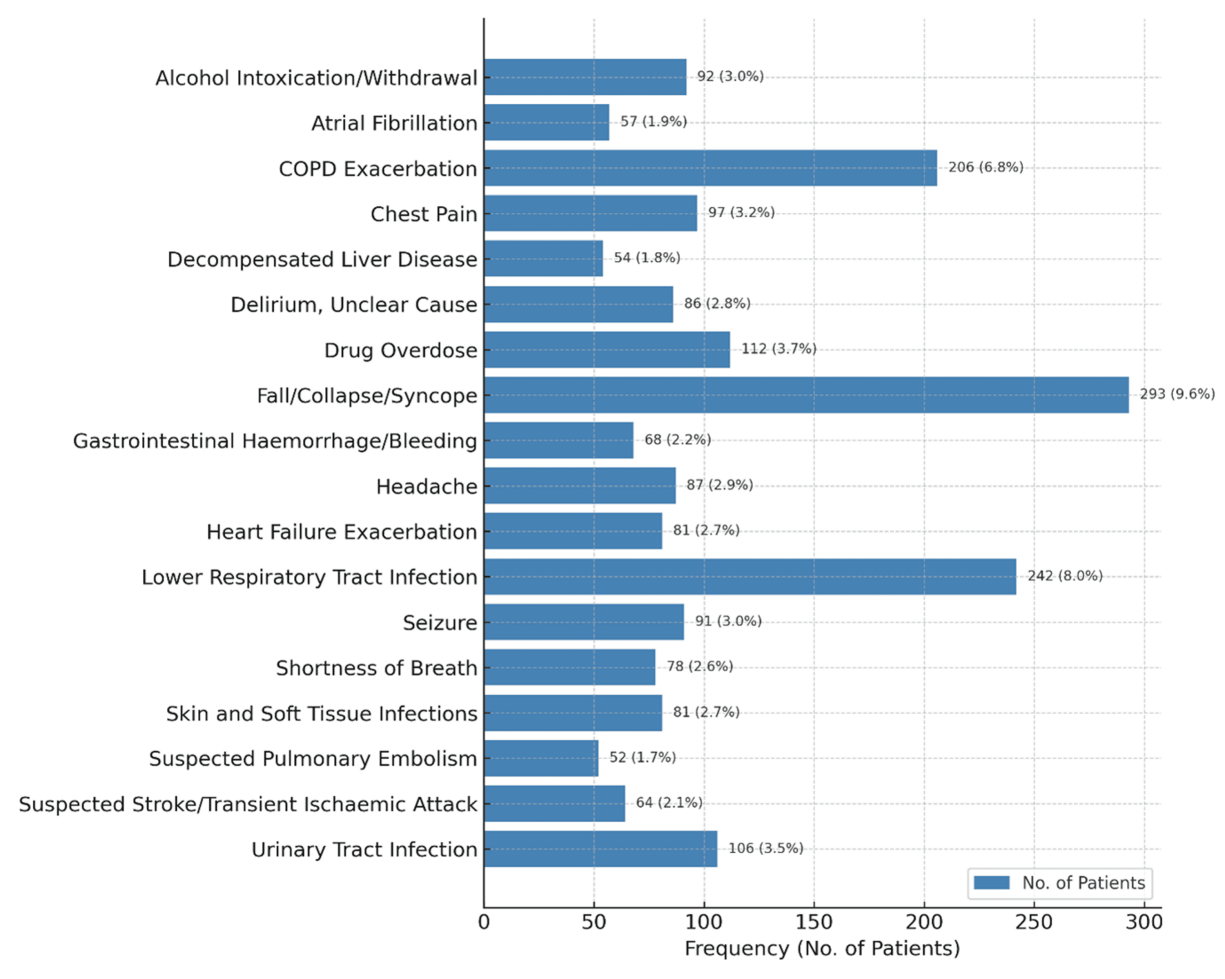
The most common reasons for admission to the acute medical admissions unit. COPD: chronic obstructive pulmonary disease

**Figure 6:**
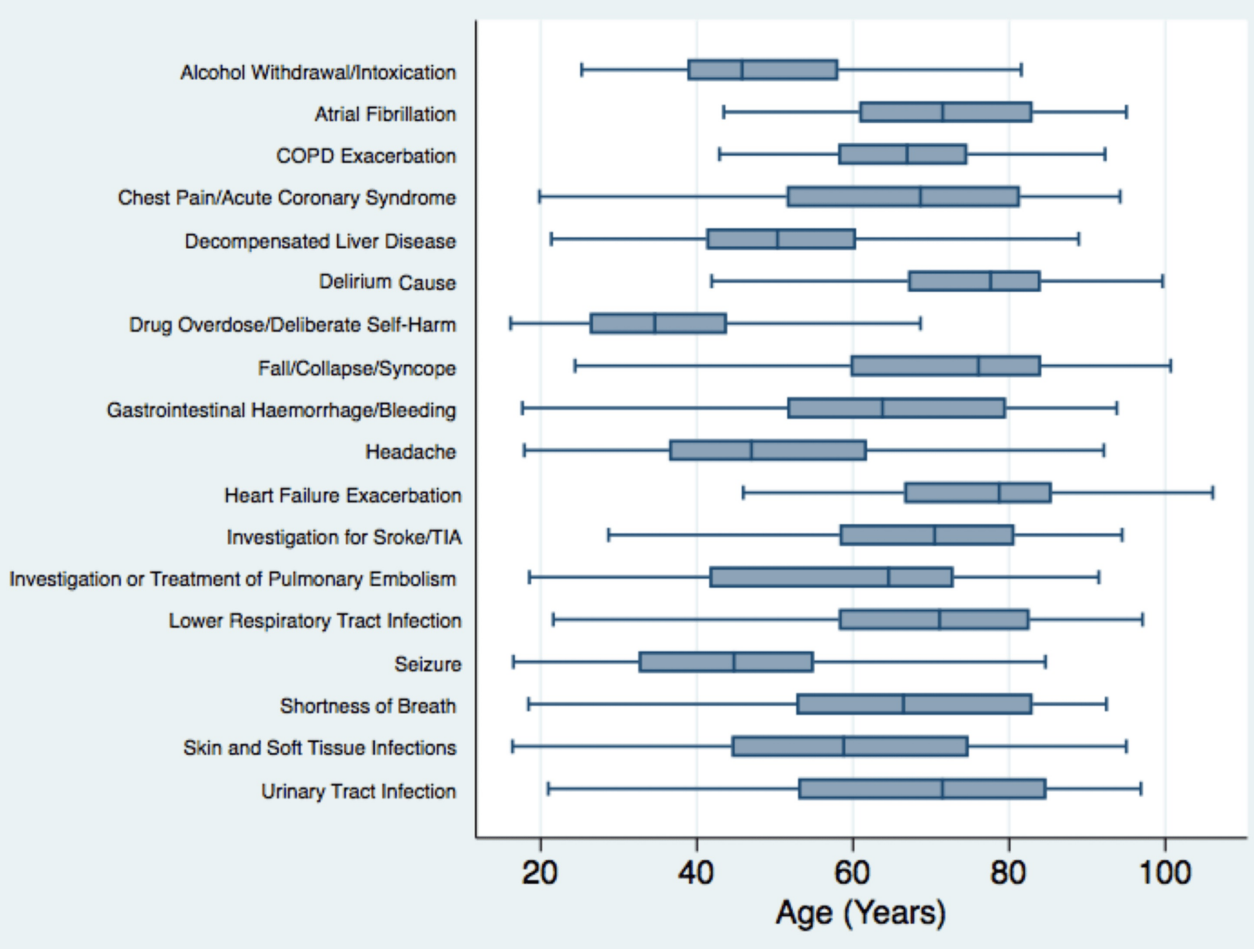
Age distribution of the most common reasons for admission. COPD: chronic obstructive pulmonary disease; TIA: transient ischemic attack

The most common reason for admission was the management and investigation of falls (with or without significant injury or fracture), which accounted for 9.32% of admissions (n=283). The other common reasons for admission were lower respiratory tract infections (242 patients, 7.97%), exacerbation of chronic obstructive pulmonary disease (206 patients, 6.78%), drug overdose (112 patients, 3.69%), and urinary tract infection (106 patients, 3.49%). The initial reasons for admission for all patients over the study period are presented in tabular format as supplementary data, broken down by age group (<50 years, 50-75 years, and >75 years) (appendix 1). The 20 most common presentations, defined as the reason for admission for at least 50 patients, affected 1,959 patients in the study (64.5%) combined and are listed with their respective age distributions in Figures 5, 6.

Immediate outcomes

The median LOS for the patients admitted to the AMAU was five days (interquartile range=2-12 days), 1,404 (46.2%) patients were admitted for five days or less, and 317 patients (10.4%) were admitted for 30 or more days.

## Discussion

Our findings reveal a substantial burden of respiratory, neurological, cardiovascular, and geriatric syndromes, underscoring the importance of skill mix and subspecialty pathways in acute medicine. Notably, the frequency of admissions for neurology and clinical toxicology is particularly striking. The data presented here can help hospitals identify the skill mix required in AMAUs and inform training curricula regarding the use of AMAUs when appropriate.

A previous study conducted in the United Kingdom attempted to characterize the initial presenting complaints and symptoms of the patients admitted to an acute medical unit [10]. For Republic of Ireland literature, we conducted a structured search of PubMed, MEDLINE, LENUS (Irish Health Repository), and Google Scholar (duration January 2000-September 2025) using Boolean terms: (“acute medical assessment unit” OR AMAU OR “acute medical admissions unit” OR AMU) AND (Ireland OR Irish) AND (“presenting complaint*” OR case mix OR diagnos* pattern* OR outcome OR admission characteristic*). We included Irish hospital studies/reports describing presentations, case mix, and/or outcomes in AMU/AMAU/MAU, adult populations, and in English. We excluded ED-only cohorts, single-condition cohorts unless dominant AMU presentations, non-Irish settings (retained for context), and editorials/opinion pieces (appendix 2).

Our search identified several Irish AMAU studies, mainly from Dublin tertiary hospitals, focusing on outcomes (mortality, LOS, readmissions) [5,11-19] and service models [3,4,20-26]. One study described older adults aged 65 years or older (appendix 3) [19]. These previous Irish AMAU studies had limited data on presenting complaints, case mix, and outcomes across the full age spectrum.

Our demographic findings, as compared to the UK national data, showed a clear picture. Our patient cohort showed a gender distribution that was highly consistent with the national average, with 49.9% of our patients being female, closely aligning with the 53% reported in the national audit [27]. However, a more nuanced story unfolded when we examined the age distribution. While our data for patients over 75 years (31.9%) initially seems higher than the 25% and 27% of patients over 80 documented in the 2023 and 2022 national audits, respectively, a closer inspection of our raw data revealed a different trend for the very old. An estimated 12.5% of our patients were over 80 years old, a figure that is a marked contrast to the 25% (2023) and 27% (2022) reported in the national audits [27]. This finding suggests that while our unit cares for a substantial number of patients aged over 75 years, it may be admitting a different case mix within this age group, with a lower proportion of the most elderly compared to the national average.

The most common reason for presentation at the AMAU in our study was the investigation and management of falls, with a median age of 77 years among those affected. By contrast, in a study of patients aged 65 years and older in the ED of another urban Irish hospital, collapse/syncope and fall/injury combined accounted for one-tenth of admissions [28]. Similarly, in the only other study conducted in an Irish AMAU examining patients aged 65 years and older, collapse was among the most common reasons for presentation [19]. Moreover, in a recent study conducted in the ED of our hospital, of 561 patients presenting with falls/syncope, unexplained falls and syncope accounted for one-quarter of all falls and were significantly associated with subsequent admission, and, overall, around 50% of falls presenting to the ED were admitted [29]. Given the significant sequelae of falls in terms of cognitive decline, gait and mobility disturbances, depression, and frailty, an emphasis on falls history (and, indeed, collateral history where appropriate) [30] and clinical workup of falls/syncope (including cardiovascular disorders) is warranted [31]. The embedding of specialist geriatric input in both the ED and AMAU is essential [32], especially given the prevalence of comorbid geriatric syndromes, such as delirium/dementia and frailty, which are common in older patients who make unscheduled ED visits [33,34]. Furthermore, the inclusion of geriatrics specialists and falls and blackout specialists in AMAUs is essential because of the prevalence of presentation in these categories, especially in aging populations. Therefore, the managers of acute hospitals may choose to assign these specialists to AMAUs or establish separate units depending on the local resources.

The reason for nearly one-quarter of all admissions in our study was classified as respiratory. This result is largely consistent with the finding in a similar UK study that one-fifth of admissions to the AMAU were respiratory cases [10] and the finding in an Irish study of patients aged 65 years and older that “difficulty breathing” accounted for nearly one-third of AMAU admissions [19]. Seizures, headaches, and delirium were the most common reasons for admission in the category of neurology, occurring at a rate more than double that reported in the UK study (8.7%) [10]. A partial explanation for this difference may be the inclusion of delirium in this category, though the number of patients admitted to our AMAU in this category (453, 14.92%) excluding delirium presentations (86, 2.83%) remains high.

A striking result of the present study is the prevalence of clinical toxicology among the patients presenting to the AMAU, almost always involving drug overdoses, alcohol intoxication, or withdrawal. The proportion of patients presenting for this reason is nearly double that found in a similar study conducted in the United Kingdom [10]. This difference may reflect the high deprivation rate in the catchment area of our hospital [35]. There are relatively few clinical toxicology experts in the United Kingdom and Ireland; therefore, acute physicians are increasingly responsible for managing this patient group. The present study highlights the need for medical curricula to emphasize the investigation and workup of this patient group in undergraduate and postgraduate medical training. Likewise, access to toxicology experts is of paramount importance in AMAUs, especially those based in urban areas where drug- and alcohol-related medical problems may be especially prevalent.

The availability of dedicated referral pathways may account for the low prevalence of hematology and oncology admissions to the AMAU. However, while pathways have been established for stroke and STEMI in our hospital, patients were frequently admitted for further investigations of suspected transient ischemic attack/stroke and chest pains by the AMAU teams. We provided data on cardiovascular and neurology presentations, and further studied the individual presenting complaints (for example, fall, headache, suspected TIA/stroke, chest pain, atrial fibrillation, heart failure, shortness of breath, and seizure) and their impact on AMAU workload. Again, this pattern of admission may reflect the prevalence of these presenting complaints to the ED.

Strengths and limitations

This study was conducted prior to the COVID-19 pandemic, which has significantly impacted the structure and dynamics of acute hospitals. While the data are similar to those collected in studies conducted after the advent of COVID-19, the fact that our study was conducted before the pandemic remains a limitation. Another limitation was that we did not include data on disease severity or comorbidities, which could further impact the length of stay. Further, although we included a variety of presentations to the AMAU over a full year, this was a single-center study, so the results may reflect local hospital structures and referral pathways. Nevertheless, this study aimed to comprehensively characterize presentations to an Irish AMAU across all age groups. Another limitation is the potential for misclassification of the nature of the initial presenting complaint, though the presenting complaint was recorded by each consultant at the post-take meeting and thus accurately reflects the initial impression and workup of the AMAU patients. Further, the level of detail in which discrete presentations were categorized is a significant strength of this study.

## Conclusions

This study describes presenting complaints, case mix, and outcomes across all adult age groups in an acute medical admissions unit (AMAU) in Ireland. This study demonstrated a wide range of presentations among the patients admitted to an Irish AMAU. The findings draw attention, in particular, to the need to include specialists in geriatric medicine and falls and blackout in AMAUs and for training in these areas. In addition, the prevalence of admissions for clinical toxicology draws attention to the need for toxicology training for AMAU staff as well as access to toxicology experts within AMAUs. Our data are essential for informing the ongoing restructuring of acute unscheduled care, the educational needs of trainees in acute medicine, and the skill mix needed by local and national providers of acute medical services.

AMAUs are specialized medical units that play a key role in the treatment of patients referred with acute medical emergencies. These facilities are structured to provide rapid, definitive assessment and management, and trials have shown shortened LOSs, improved patient outcomes, and more efficient use of hospital resources. However, more data are needed to inform the strategic planning of healthcare facilities, the structuring of training programs for medical trainees, and the skill mix needed in AMAUs. Furthermore, the data on LOS, the range of age groups, and clinical diagnosis are valuable for informing the provision of services, the strategic planning of AMAUs, and the range of training opportunities for trainees in medical specialties.

## Figures and Tables

**Figure 7:**
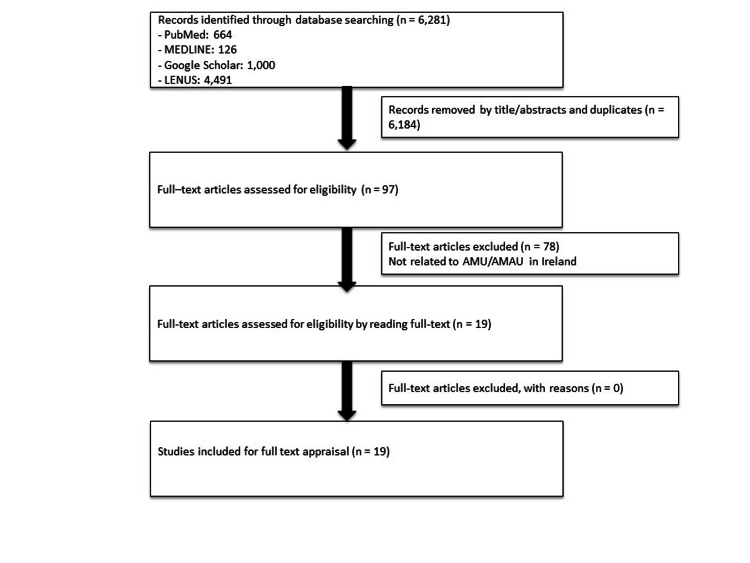
PRISMA flow diagram illustrating the process of study identification, screening, eligibility, and inclusion. n: no. of patients; PRISMA: Preferred Reporting Items for Systematic Reviews and Meta-Analyses; AMU: acute medical unit; AMAU: acute medical admissions unit

## Appendices

Appendix 1

**Table 1:** Irish AMAU and related studies. AMAU: acute medical admissions unit

Studies	Location	Population/focus	Main outcomes	Limitations
Conway et al. 2018 (Ir J Med Sci) [5]	Dublin	15-year AMAU follow-up	Sustained improved outcomes, shorter LOS	Single-center
Rooney et al. (QJM) 2008 [11]	Dublin, AMAU	5-year prospective	Reduced hospital mortality post-AMAU	No presenting complaint data
Glynn et al. (Clin Med {Lond}) 2011 [12]	Dublin, national	Emergency medical readmissions	Long-term readmission trends; mortality burden	Administrative data; not AMAU-specific
Conway et al. 2014 (QJM) [13]	Dublin	10-year AMAU outcomes	Improved long-term outcomes	Mortality focus; no case mix
Conway et al. 2018 (Ir J Med Sci) [14]	Dublin	Weekend emergency admissions	Mortality improved over time	No presenting complaints
Conway et al. 2019 (Eur J Intern Med) [15]	Dublin	Extended AMAU cohort	Readmission, LOS, mortality trends	No complaint-level data
Conway et al. 2023 (QJM) [16]	Dublin	Acute medical admissions	Short- and long-term mortality analysis	No presenting complaints
Conway et al. 2023 (Ir J Med Sci) [17]	Dublin	Admissions by specialty	Mortality and LOS differed by on-call specialty	Retrospective, single site
Conway et al. 2024 (Ir J Med Sci) [18]	Dublin	Weekend vs. weekday	Lower 30-day mortality for weekend admissions, but higher long-term mortality	Retrospective
Fallon et al. 2015 (Ir Med J) [19]	Irish AMAU	≥65 years	Frailty and falls are common; poor outcomes	Older subgroup only
Moloney et al. (Emerg Med J) 2006 [3]	Dublin	AMAU service audit	Quality indicators improved (funnel plots)	Process focus
Coary et al. (Acute Med) 2014 [4]	Dublin	12 years’ AMAU	Mortality reduced via AMAU admission pathway	No complaint data
Moloney et al. (Emerg Med J) 2006 [20]	Dublin, ED interface	ED patients awaiting beds	Showed admission delays pre-AMAU	Not patient-level
Watts et al. (Ir Med J) 2011 [21]	Ireland, multisite	AMAU model	AMAUs efficient alternative to ward-based care	Service-level only
Subbe et al. (Eur J Intern Med) 2014 [22]	Multicentre (Ireland/UK)	Triage feasibility	Reduced LOS; cost-effective	Pilot; multicountry
Sheahan 2001 [23]	Irish General Hospital	Early MAU evaluation	Operational feasibility	Small scale
Acute medical units 2004 [24]	National (Ireland)	Policy document	Recommended AMU establishment	Grey literature
Chantal 2015 [25]	Cork University Hospital	Local AMU audit	Improved ED flow post-AMU	Local scope only
Report of the National Acute Medicine Programme 2010 [26]	National	National program	Standardized AMU model of care	No outcomes

Appendix 2

Appendix 3 

**Table 2:** Reasons for admission in patients attending the acute medical admissions unit. n: no. of patients (percentage displayed where n >10); COPD: chronic obstructive pulmonary disease; TIA: transient ischemic attack; DKA: diabetic ketoacidosis; DVT: deep vein thrombosis

Category	Reason for admission	N (%)	Median age (range)	<50 years	50-75 years	>75 years
Cardiovascular	Chest pain/acute coronary syndrome	97 (3.2%)	69.7 (19.8-94.2)	20 (21.1%)	36 (37.9%)	39 (41.0%)
	Heart failure exacerbation	81 (2.7%)	78.7 (35-106)	2 (2.5%)	28 (34.6%)	51 (63.0%)
	Atrial fibrillation	57 (1.9%)	71.5 (43.5-95)	2 (3.5%)	31 (54.4%)	24 (42.1%)
	Other arrhythmia	25 (0.8%)	73.3 (32.5-86.4)	4 (16%)	9 (36%)	12 (48%)
	Hypertension	6 (0.2%)	68.1 (61.1-81.3)	0	5	1
	Endocarditis	4 (0.1%)	47.9 (31.8-64.8)	2	2	0
	Pericarditis	4 (0.1%)	56.9 (47.1-78.4)	1	2	1
	Ischemic limb	3 (0.1%)	72.3 (60.5-94.5)	0	2	1
	Aneurysm	1 (0.03%)	94	0	0	1
Respiratory	Lower respiratory tract infection	242 (8.0%)	71.1 (19.2-97.1)	47 (19.4%)	92 (38.0%)	103 (42.6%)
	COPD exacerbation	206 (6.8%)	66.8 (29.3-92.3)	15 (7.3%)	144 (69.9%)	47 (22.8%)
	Shortness of breath	78 (2.6%)	66.5 (18.4-92.5)	11 (14.1%)	36 (46.2%)	31 (39.7%)
	Pulmonary embolism	52 (1.7%)	64.5 (18.6-91.5)	16 (30.8%)	25 (48.1%)	11 (21.2%)
	Asthma	47 (1.6%)	46.3 (18.7-87.7)	29 (61.7%)	12 (25.5%)	6 (12.8%)
	Pleural effusion	23 (0.8%)	81 (45.2-96.8)	2 (8.7%)	5 (21.7%)	16 (69.6%)
	Pneumothorax	13 (0.4%)	33.8 (18.6-65.6)	11 (84.6%)	2 (15.4%)	0
	Fibrosis	13 (0.4%)	73 (58-90.1)	0	2 (15.4%)	11 (84.6%)
	Cough	13 (0.4%)	53 (32-80.2)	6 (46.2%)	4 (30.8%)	3 (23.1%)
Neurology	Seizure	91 (3.0%)	45 (16.5-84.7)	58 (63.7%)	29 (31.9%)	4 (4.4%)
	Headache	87 (2.9%)	47 (17.9-92.1)	46 (52.9%)	31 (35.6%)	10 (11.5%)
	Delirium/confusion	86 (2.8%)	76 (29-99.6)	12 (14.0%)	23 (26.7%)	39 (45.4%)
	Stroke/TIA (investigation)	64 (2.1%)	70 (28.7-94.5)	3 (4.7%)	32 (50%)	29 (45.3%)
Renal	Urinary tract infection	106 (3.5%)	71.5 (21-96.9)	25 (23.6%)	31 (29.4%)	50 (47.2%)
	Acute kidney injury	38 (1.3%)	65.8 (36-95.7)	7 (18.4%)	17 (44.7%)	14 (36.8%)
Clinical toxicology	Drug overdose	112 (3.7%)	34.6 (16-74)	93 (83.0%)	19 (16.9%)	0
	Alcohol withdrawal/intoxication	92 (3.0%)	45.8 (25.2-81.6)	50 (54.4%)	37 (40.2%)	5 (5.4%)
Endocrine and metabolic	Uncontrolled diabetes/DKA	33 (1.1%)	39.5 (16-93.5)	19 (57.6%)	9 (27.3%)	5 (15.2%)
	Hyponatremia	20 (0.7%)	68 (48.6-89.2)	1 (5.0%)	12 (50.0%)	9 (45.0%)
Dermatology	Skin and soft tissue infection	81 (2.7%)	58.8 (16.4-95)	26 (32.1%)	35 (43.2%)	20 (24.7%)
	Leg or foot ulcers	14 (0.5%)	63.4 (29.2-82.7)	6 (37.5%)	4 (28.6%)	4 (28.6%)
Hematology	DVT investigation	43 (1.4%)	63 (24.7-93.9)	20 (46.5%)	9 (20.9%)	14 (32.6%)
	Symptomatic anemia	31 (1.0%)	70.5 (22.1-91.9)	8 (25.8%)	13 (41.9%)	10 (32.3%)
Infectious diseases	Pyrexia of unknown source	46 (1.5%)	53.2 (17.3-92.9)	19 (41.3%)	15 (32.6%)	12 (26.1%)
	Abscess	10 (0.3%)	33 (23.3-74.8)	7	3	0
Oncology and palliative care	Investigation for new/progression	36 (1.2%)	65.6 (32.6-88)	5 (13.9%)	20 (55.6%)	11 (30.6%)
Rheumatology	Back and neck pain	29 (1.0%)	66 (21.3-73.3)	10 (34.5%)	5 (17.2%)	14 (48.3%)
	Joint pain	20 (0.7%)	69.2 (24.8-90.6)	8 (40.0%)	4 (20.0%)	4 (20.0%)
Psychiatry	Dementia	9 (0.3%)	82 (79.3-90.3)	0	0	9
	Delusions/psychosis	5 (0.2%)	42 (19.2-77)	3	1	1
Other/undifferentiated	Nausea/vomiting	26 (0.9%)	62.7 (26.4-90.6)	8 (30.8%)	9 (34.6%)	9 (34.6%)
	Generally unwell	12 (0.4%)	80 (42.9-88.6)	1 (8.3%)	2 (16.7%)	9 (75.0%)
